# Incidence of giant cell arteritis in Western Norway 1972–2012: a retrospective cohort study

**DOI:** 10.1186/s13075-017-1479-6

**Published:** 2017-12-15

**Authors:** L. K. Brekke, A. P. Diamantopoulos, B-T. Fevang, J. Aβmus, E. Esperø, C. G. Gjesdal

**Affiliations:** 1 Hospital for Rheumatic Diseases, Haugesund, Norway; 2 Department of Clinical Science, University of Bergen, Bergen, Norway; 3 Bergen Group of Epidemiology and Biomarkers in Rheumatic Disease (BEaBIRD), Department of Rheumatology, Haukeland University Hospital, Bergen, Norway; 4 Martina Hansens Hospital, Bærum, Norway; 5 Centre for Clinical Research, Haukeland University Hospital, Bergen, Norway; 6 Hospital for Rheumatic Diseases (HSR AS), PB 2175, 5504 Haugesund, Norway

**Keywords:** Giant cell arteritis, Temporal arteritis, Incidence, Epidemiology, Vasculitis, Temporal artery biopsy, Norway

## Abstract

**Background:**

Giant cell arteritis (GCA) is the most common systemic vasculitis in persons older than 50 years. The highest incidence rates of the disease have been reported in Scandinavian countries. Our objective was to determine the epidemiology of GCA in an expected high-incidence region during a 41-year period.

**Methods:**

This is a hospital-based, retrospective, cohort study. Patients diagnosed with GCA in Bergen health area during 1972–2012 were identified through computerized hospital records (n = 1341). Clinical information was extracted from patients’ medical journals, which were reviewed by a standardized method. We excluded patients if data were unavailable (n = 253), if the reviewing rheumatologist found GCA to be an implausible diagnosis (n = 207) or if the American College of Rheumatology (ACR) 1990 classification criteria for GCA were not fulfilled (n = 89). Descriptive methods were used to characterize the sample. Incidence was analyzed by graphical methods and Poisson regression.

**Results:**

A total of 792 patients were included. The average annual cumulative incidence of GCA was 16.7 (95% CI 15.5-18.0) per 100,000 of the population ≥ 50 years old. The corresponding incidence for biopsy-verified GCA was 11.2 (95% CI 10.2–12.3). The annual cumulative incidence increased with time in the period 1972–1992 (relative risk (RR) 1.1, *p* < 0.001) but not in 1993–2012 (RR 1.0, *p* = 0.543). The incidence was higher in women compared to men (average annual incidence 37.7 (95% CI 35.8–39.6) vs. 14.3 (95% CI 13.2–15.5), *p* < 0.001) with women having a twofold to threefold higher incidence rate throughout the study period. Average annual incidence increased with age until the 7th decade of life in both sexes throughout the study period (2.8 (95% CI 2.3–3.3) for age <60, 15.5 (95% CI 14.4–16.8) for age 60–69, 34.5 (95% CI 32.8–36.4) for age 70–79 and 26.8 (95% CI 25.3-28.4) for age ≥80 years, *p* < 0.001 for all age adjustments).

**Conclusions:**

Our study confirms an incidence of GCA comparable to previous reports on Scandinavian populations. Our results show increasing incidence from 1972 through 1992, after which the incidence has levelled out.

**Electronic supplementary material:**

The online version of this article (doi:10.1186/s13075-017-1479-6) contains supplementary material, which is available to authorized users.

## Background

Giant cell arteritis (GCA) is the most common systemic vasculitis in adults [1–4]. It affects primarily the medium and large arteries, almost exclusively in persons over the age of 50 years and more commonly affects women than men [1, 4–10]. The disease occurs most frequently in populations of northern Europe, and in particular in Scandinavian countries where the incidence has been reported as high as 32.8 per 100,000 inhabitants over the age of 50 years [7, 9, 11–14]. The etiology of GCA is not fully understood although it is clear that hyper-reactivity of the immune system plays a critical role, perhaps triggered by an infection or toxin in a person with genetic risk factors [15–19]. Temporal artery biopsy (TAB) remains the standard for definitive diagnosis, but suffers from low sensitivity [20, 21]. Biopsy may not be necessary in patients with typical disease features accompanied by characteristic imaging findings [1, 15].

GCA may be associated with life-threatening complications [22–26]. Most recent studies have nevertheless concluded that the disease has little or no adverse impact on patients’ mortality, but worldwide burden and costs of GCA are large and increasing [14, 16, 27, 28]. The optimal follow-up regimen for patients undergoing treatment for GCA is not currently established [15, 16]. Accurate and up-to-date knowledge of the epidemiology of this disease is therefore of great importance. There are few recently published studies of trends in incidence in large cohorts of people with GCA [9, 14, 29]. A 14-year epidemiologic study including 840 patients from Sweden was published in 2015 [29]. Before that, no updated epidemiologic data on GCA had been published from northern Europe since the 1990s despite potential alterations in environmental or lifestyle exposures and ethnic/genetic composition of the population.

Thus, the aim of our study is to broaden current knowledge about the epidemiology of GCA. The long-term trend of GCA incidence in Norway has never before been published. This study spans a 41-year period. To the best of our knowledge this is longer than any prior epidemiologic study on GCA originating from Europe.

## Methods

This is a retrospective, observational, cohort study. Our material represents a predominantly Caucasian referral cohort from mixed rural and urban areas. The study setting was Bergen health area, consisting of three somatic hospitals: Haukeland University Hospital, Haraldsplass Deaconess Hospital and Voss Hospital. Together, these hospitals have the responsibility to provide specialist health care services to the inhabitants of 22 municipalities (Bergen and surrounding area) in Hordaland county in Western Norway. In this region there is only one laboratory for pathology, and no private hospitals that give care to rheumatology patients. Patients with suspected vasculitis are referred to a hospital department, but the choice of referral department may vary depending on the presenting clinical features of each case. Patients with polymyalgia rheumatica (PMR) who have inadequate response to glucocorticoid treatment, and elderly patients with fever or prolonged constitutional symptoms are also referred for assessment of GCA as a differential diagnosis. The majority (>90%) of rheumatologists and internal medicine specialists in the area are hospital-based, with only three private rheumatologists in the region, all publicly funded and collaborating closely with the hospital departments. This ensures a high capture-rate of incident cases in our study. We collected data on patients registered with the diagnosis of GCA following an outpatient visit or admission to any ward in one of the three study hospitals between 1 January 1972 and 31 December 2012 (41-year period). The International Classification of Diseases (ICD) coding system was used to identify patients from the hospitals’ electronic administrative patient records, ICD-8 (446.4) for 1972–1987, ICD-9 (446.5) for 1987–1998 and ICD-10 (M31.5-6) for 1999–2012. A consultant rheumatologist or an experienced rheumatology fellow reviewed the patients’ records and recorded clinical details on a standardized form (see Additional file 1). We excluded patients if their GCA diagnosis originated prior to the beginning of our study, if data were unavailable, if the review of records concluded that GCA was an implausible diagnosis or if the American College of Rheumatology (ACR) 1990 classification criteria for GCA were not fulfilled. However, patients with a clinically appropriate GCA diagnosis, though not fulfilling the criteria, were included in a sub-analysis. For the computing of incidence we also excluded patients not residing in one of the 22 municipalities primarily served by a Bergen health area hospital. The patient’s residential address at time of diagnosis was obtained from the population register in Norway, which is run by the Norwegian Tax Administration. The background population data were obtained from Statistics Norway (www.ssb.no). We used all inhabitants over the age of 50 years residing in one of the 22 municipalities in Bergen health area. For incidence stratified after sex and age groups (<60 years, 60–69 years, 70–79 years and 80+ years), the corresponding subpopulations were used as persons at risk. For the subgroups related to biopsy and erythrocyte sedimentation rate (ESR), we used the entire background population. The original Westergren method, the gold standard for the determination of the ESR, was used for the period 1972–1987. Newer methods for the measurement of ESR were used for the remainder of the study period (Seditainer^TM^ 1987–1997, Sedisystem^TM^ 1997–2007 and SEDI-15^TM^ 2007–2012). The newer methods were all validated and shown to correlate reasonably well with the original Westergren method, but studies have also shown that there may be significant differences to the Westergren method at higher values (i.e. underestimating the ESR) [30–32].

### Statistical analysis

Descriptive methods were used to characterize the sample. The *t* test was used for comparing continuous variables and the chi-square or Fisher’s exact test for comparing categorical variables. Annual cumulative incidence, i.e. annual number of diagnoses divided by annual number of persons at risk (reported per 100,000 [33]) was calculated both for the entire patient group and for groups stratified by sex, age group, biopsy result and ESR. Incidence was analyzed by graphical methods and Poisson regression. The annual cumulative incidence was plotted in the time domain both raw and smoothed by the moving average of 5 years, completed by the mean of all annual incidences with the 95% confidence interval (CI) based on the Poisson distribution. Inference was by regression models for the annual cumulative incidence depending on time, i.e. year of appearance (unadjusted), time and sex, time and age, and time and ESR at time of diagnosis. These models were computed for the entire observation interval and separately for the time periods 1972–1992 and 1993–2012. The year 1992 was chosen as the cutoff to divide our study period into two intervals of equal duration. This cutoff also provided an opportunity to investigate the impact of new classification criteria for GCA published in 1990, assuming there might be a 1–2 year delay before widespread clinical implementation. The general significance level was set to 0.05. The computing was done using the Statistical Package for the Social Sciences (SPSS) software version 24 (IBM Corp, Armonk) and R software version 3.4 [34]. Graphics were created using Matlab 9.0 (Mathworks Inc., Natick).

## Results

**Fig. 1:**
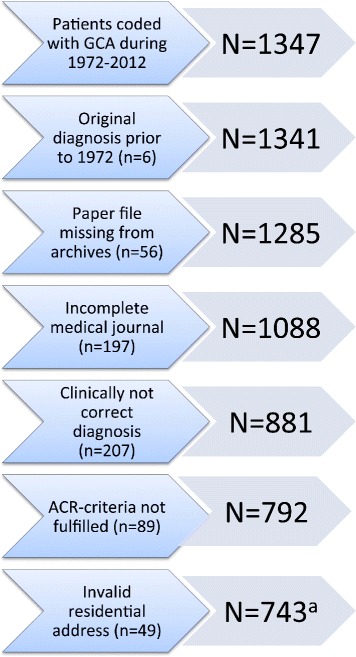
Results of the search for cases of giant cell arteritis (GCA) in Bergen health area 1972–2012. *n* refers to numbers of excluded patients and *N* refers to the remaining cohort. ^a^Of the 792 identified patients, 49 had a residential address in a municipality not primarily served by one of the study hospitals

The patient inclusion process is presented in Fig. 1. A total of 1347 patients were registered with the diagnosis of GCA during the study period. Of these, 555 were excluded from all analyses, and an additional 49 were excluded from computation of incidence. Thus, for the main analyses we included 792 individuals, 566 (71.5%) women and 226 (28.5%) men. Mean age at onset was higher in women (73.5 years (SD 8) vs. 72.1 years (SD 9), *p* = 0.041), who also had increased risk of presenting with polymyalgia rheumatic (PMR) compared to men (*p* = 0.008) (Table 1). Apart from that there were no significant differences between sexes. There were 528 patients (66.7%) with a positive TAB and 180 patients (22.7%) with a negative TAB. For the remaining 84 patients (10.6%) TAB was not performed or biopsy results were inconclusively or insufficiently reported. Patient characteristics are presented in Table 1.

**Table 1:** Characteristics of the study population

Clinical characteristic		Overall *N* = 792	Female *N* = 566	Male *N* = 226	*P* value for difference between sexes
Mean age at onset of GCA, years (SD)		73.1 (8)	73.5 (8)	72.1 (9)	0.041
ACR criteria fulfilled, persons, *n* (%)		792 (100)	566 (100)	226 (100)	
Age ≥50 years at disease onset, *n* (%)		788 (99.5)	564 (99.6)	224 (99.1)	0.322 (Fisher)
New onset headache, *n* (%)		573 (72.3)	405 (71.6)	168 (74.3)	0.897
Temporal artery tenderness, *n* (%)		365 (46.1)	256 (45.2)	109 (48.2)	0.390
Decreased temporal pulse, *n* (%)		227 (28.7)	168 (29.7)	59 (26.1)	0.416
ESR ≥50 mm/h, *n* (%)		717 (90.5)	510 (90.1)	207 (91.6)	0.351
Biopsy showing vasculitis, *n* (%)		528 (66.7)	378 (66.8)	150 (66.4)	0.984
Giant cells in biopsy, *n* (%)		243 (30.7)	185 (32.7)	58 (25.7)	0.137
Jaw claudication, *n* (%)		181 (22.9)	134 (23.7)	47 (20.8)	0.763
Polymyalgia rheumatica, *n* (%)		242 (30.6)	192 (33.9)	50 (22.1)	0.008
Peripheral arthritis, *n* (%)		32 (4.0)	23 (4.1)	9 (4.0)	0.760
Visual disturbance, *n* (%)		146 (18.4)	103 (18.2)	43 (19.0)	0.786
Blindness in one or both eyes, *n* (%)		32 (4.0)	23 (4.1)	9 (4.0)	0.958
Scalp necrosis, *n* (%)		6 (0.8)	4 (0.7)	2 (0.9)	0.679 (Fisher)
Mean ESR, mm/h (SD)	N = 782^a^	84.6 (27)	84.2 (28)	85.7 (27)	0.483
Mean CRP, mg/L (SD)	N = 601^a^	91.2 (63)	88.3 (62)	99.2 (65.3)	0.059

If a given variable was not documented in the patient’s record it was registered as missing. In subsequent statistical analyses missing data were treated as negative findings. The *t* test was used for comparing continuous variables and the chi-square or Fisher’s exact test for comparing categorical variables.

*GCA* giant cell arteritis, *ACR* American College of Rheumatology, *ESR* erythrocyte sedimentation rate, *CRP* C-reactive protein

^a^Mean laboratory values were calculated within the subset with available data

**Fig. 2:**
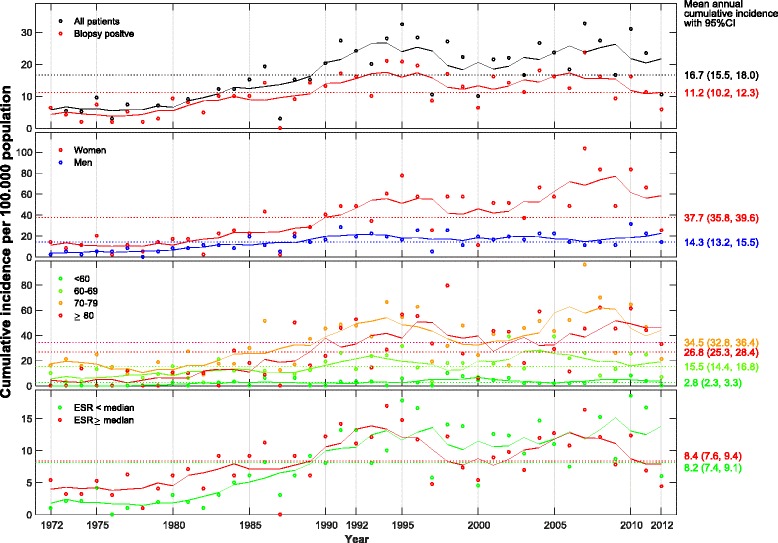
Annual cumulative incidence of giant cell arteritis (American College of Rheumatology (ACR) criteria fulfilled) in Bergen health area 1972–2012. Overall and ESR-specific cumulative incidence calculated as cases per 100,000 general population over the age of 50 years. Incidence by sex was calculated per 100,000 women or men, respectively, and incidence by the different age categories was calculated per 100,000 population of the same age categories (<60 years, 60–69 years, 70–79 years and 80+ years). Points plotted represent raw incidence. Solid lines were estimated using the smoothing technique of a moving average of 5 years. ESR, erythrocyte sedimentation rate

**Fig. 3:**
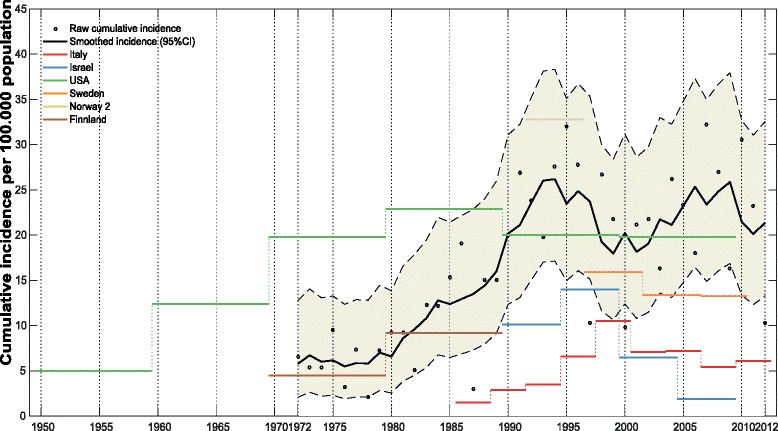
Comparison of giant cell arteritis (GCA) incidence in our cohort with reported incidence from adjacent countries and key studies on GCA time trends. The mean annual cumulative incidence in the present study is shown as a moving average of 5 years. The mean annual incidences from other studies are shown as several means for separate time intervals of varying length. Data chosen for comparison are from Italy 1986–2012 [14], Israel 1990–2009 [39], Minnesota (USA) 1950–2009 [9], Finland 1970–1989 [46], Sweden 1997–2010 [29] and Norway 1992–1996 [11]

The average annual cumulative incidence of GCA per 100,000 population over the age of 50 years was 16.7 (95% CI 15.5–18.0). The corresponding incidence for biopsy-verified GCA was 11.2 (95% CI 10.2–12.3). The cumulative incidence increased with time in the period 1972–1992 (relative risk (RR) 1.1, *p* < 0.001) but not for 1993–2012 (RR 1.0, *p* = 0.543) (Table 2). The highest annual incidence observed was 32.8 in 2007 and the lowest was 2.1 in 1978 (Fig. 2). The incidence was higher in women compared to men (average annual incidence 37.7 (95% CI 35.8–39.6) vs. 14.3 (95% CI 13.2–15.5), *p* < 0.001) with women having a twofold to threefold higher cumulative incidence throughout the study period (Fig. 2 and Table 2). Incidence increased with age until the 7th decade of life in both sexes throughout the study period (average annual incidence 2.8 (95% CI 2.3–3.3) for age <60, 15.5 (95% CI 14.4–16.8) for age 60–69, 34.5 (95% CI 32.8–36.4) for age 70–79 and 26.8 (95% CI 25.3-28.4) for age ≥80 years, *p* < 0.001 for all age adjustments) (Table 2). Fifty percent of patients presented with ESR >85 mm/h and from 1972 to 1992 we observed a significant increase in incident cases of patients presenting with ESR above the median of 85 (RR 1.4, *p* < 0.006). This was not observed in the time period 1993–2012 (RR 0.9, *p* = 0.116). The overall annual cumulative incidence and sex-specific, age-specific and ESR-specific effects on incidence are presented in Fig. 2. Figure 3 and Table 3 show the mean annual cumulative incidence of our cohort alongside the mean incidences reported in other populations. Our results are comparable to previous reports from Scandinavian populations and to key studies of long-term trends in GCA incidence.

**Table 2:** The incidence of giant cell arteritis (GCA) in Bergen health area 1972–2012

a Mean annual cumulative incidence						
	All time		1972–1992		1993–2012	
	Cumulative incidence	95% CI	Cumulative incidence	95% CI	Cumulative incidence	95% CI
All patients	16.7	(15.5, 18.0)	11.2	(9.8, 12.7)	22.5	(20.5, 24.7)
Sex						
Female	37.7	(35.8, 39.6)	20.7	(18.8, 22.7)	55.4	(52.2, 58.8)
Male	14.3	(13.2, 15.5)	10.5	(9.2, 12.0)	18.3	(16.5, 20.3)
Age, years						
<60	2.8	(2.3, 3.3)	1.5	(1.0, 2.1)	4.1	(3.3, 5.0)
60–69	15.5	(14.4, 16.8)	10.9	(9.6, 12.4)	20.3	(18.4, 22.4)
70–79	34.5	(32.8, 36.4)	23.4	(21.4, 25.6)	46.2	(43.3, 49.2)
80+	26.8	(25.3, 28.4)	14.3	(12.8, 16.0)	39.9	(37.2, 42.7)
ESR, mm/h						
ESR <85	8.2	(7.3, 9.1)	4.5	(3.7, 5.5)	12.0	(10.6, 13.6)
ESR >85	8.4	(7.6, 9.3)	6.6	(5.5, 7.7)	10.4	(9.0, 11.8)
b Relative risk (RR) according to time, sex, age and ESR						
	1972–1992			1993–2012		
	RR	95% CI	*p* value	RR	95% CI	*p* value
Unadjusted						
Time, years	1.1	(1.1, 1.1)	<0.001	1.0	(1.0, 1.0)	0.543
Sex						
Time, years)	1.1	(1.1, 1.1)	<0.001	1.0	(1.0, 1.0)	0.014^a^
Sex (male vs. female)	0.5	(0.4, 0.6)	<0.001	0.3	(0.3, 0.4)	<0.001
Age						
Time, years	1.1	(1.1, 1.1)	<0.001	1.0	(1.0, 1.0)	0.135
60–69 vs. <60	7.2	(5.1, 10.6)	<0.001	5.0	(3.9, 6.4)	<0.001
70–79 vs. <60	15.4	(11.0, 22.5)	<0.001	11.3	(9.1, 14.3)	<0.001
80+ vs. <60	9.5	(6.7, 13.9)	<0.001	9.8	(7.8, 12.4)	<0.001
ESR						
Time, years	1.1	(1.1, 1.1)	<0.001	1.0	(1.0, 1.0)	0.632
ESR, >median vs. <median	1.4	(1.1, 1.9)	0.006	0.9	(0.7, 1.0)	0.116

Overall and erythrocyte sedimentation rate (ESR)-specific cumulative incidence reported as cases per 100,000 background population over the age of 50 years. Incidence for sex reported per 100,000 women or men, respectively, and incidence for the different age categories reported per 100,000 population of the same age categories (<60 years, 60–69 years, 70–79 years and 80+ years). Relative risk calculated according to Poisson regression models for the two time periods 1972–1992 and 1993–2012. *CI* confidence interval

^a^The RR changed significantly, but the magnitude of the change was too small to visualize with rounding to one decimal place: 1972–1992, RR 1.091 (95% CI 1.076–1.106); 1993–2012, RR 1.011 (95% CI 1.002–1.020)

**Table 3:** The incidence of giant cell arteritis (GCA) in various populations and key features of underlying epidemiologic studies

Location (reference)	Time period	Inclusion criteria	Number of subjects (N)		Annual incidence^a^	
			Biopsy-proven only	All cases^b^	Biopsy-proven only	All cases^b^
Norway (PS)	1972–2012	ACR 1990 criteria	528	792	11.2	16.7
Norway [11]	1992–1996	Clinical diagnosis	47	53	29.1	32.8
Norway [13]	1987–1994	Biopsy-proven only	66	NR	29.0	NR
Sweden [29]	1997–2010	Biopsy-proven only	840	NR	14.1	NR
Sweden [36]	1976–1995	Biopsy-proven only	665	NR	22.2	NR
Sweden [47]	1973–1975	Clinical diagnosis	74	126	16.8	28.6
Finland [46]	1969–1989	Biopsy-proven only	66	NR	7.2	NR
Denmark [38]	1982–1994	Clinical diagnosis	NR	NR	15.1	20.4
Iceland [7]	1984–1990	Clinical diagnosis	125	133	25.4	27.0
Minnesota, USA [9]	2000–2009	ACR 1990 criteria + radiologic criteria^c^	56	74	NR	19.8
New Zealand [6]	1996–2005	Biopsy-proven only	70	NR	12.7	NR
Israel [39]	1990–2009	ACR 1990 criteria	NR	140	NR	8.1
Italy [14]	1986–2012	Biopsy-proven only	285	NR	5.8	NR
Spain [40]	1981–2005	Biopsy-proven only	255	NR	10.1	NR
Turkey [41]	2002–2008	Clinical diagnosis	13	19	NR	1.1

*PS* present study, *NR* not reported, *ACR* American College of Rheumatology

^a^Mean annual incidence reported as cases per 100,000 population age ≥50 years

^b^Including probable cases based on clinical diagnosis despite negative biopsy or in patients in whom biopsy was not performed

^c^Seven patients were included based on radiologic criteria. These were all ≥50 years old with elevated erythrocyte sedimentation rate or C-reactive protein, and evidence of large-vessel vasculitis on angiographic computed tomography, angiographic magnetic resonance imaging or positron emission tomography

We also performed the analyses for the extended cohort of 881 patients who were clinically appropriately diagnosed as having GCA in the study period, regardless of fulfillment of ACR criteria. This resulted in a higher annual incidence of GCA (overall 18.4, female 41.2, male 15.9), but there were no differences between trends in incidence, nor in the clinical characteristics of this cohort compared to the 792 patients who fulfilled the ACR criteria. Among the 89 patients who were considered appropriately diagnosed with GCA despite not fulfilling the ACR 1990 criteria, 11 had a biopsy result at time of diagnosis that showed cranial arteritis and 1 patient was later proven to have giant cell arteritis on autopsy. Evaluating the 89 patients according to the expansion of the 1990 ACR criteria for GCA proposed by Dejaco et al., we classified 53 of these patients (59.6%) as having GCA [35]. The presence of PMR was the “new item” that changed the classification status in 25 of the patients. In the remaining patients the new item was C-reactive protein (CRP) level (nine patients), visual symptoms or visual loss (seven patients), jaw claudication (two patients), prolonged fever (two patients), biopsy result (one patient) or a combination of multiple new items (seven patients).

A total of 253 patients were excluded on account of missing or incomplete medical records. Of these, 168 (66.4%) were female and 85 (33.6%) male. Their mean age at time of first registration with the diagnosis was 71.3 years (SD 13). If they had all fulfilled the inclusion criteria of our study we would have observed an average annual incidence of GCA of 24.5 per 100,000 population over the age of 50 years. The cases excluded due to missing or incomplete records were evenly distributed throughout the study period with a peak in the early 1990s. The majority of these cases (45.8%) were registered with the diagnosis of GCA by an internal medicine department, including departments for cardiac (nine cases), pulmonary (six cases) and neurological (fifteen cases) diseases. The ophthalmology department registered 29.2%, and the rheumatology department registered 18.2% of the cases with missing data. The remaining 6.7% (17 cases) were registered with the diagnosis of GCA by the following departments: general surgery (seven cases), plastic surgery (one case), orthopaedic (one case), urology (one case), dermatology (two cases), ear nose and throat (one case), geriatric (one case), oncology (two cases) and physical medicine and rehabilitation (one case).

## Discussion

In our large referral cohort from 1972 to 2012, the mean annual cumulative incidence of GCA fulfilling the ACR 1990 criteria was 16.7 per 100,000 persons aged 50 years or more. The mean annual incidence in all patients clinically diagnosed as having GCA, regardless of fulfillment of ACR criteria, was 18.4 per 100,000, whereas the incidence of biopsy-verified GCA only was 11.2 per 100,000 persons aged 50 years or more. These rates may appear low compared to previously reported incidence of GCA in Scandinavian populations [7, 11, 13, 29, 36–38]. However, looking at our mean annual incidence for shorter time periods than the overall 41-year period, the results are in line with previous reports. Reported incidence rates from the 1980s and 1990s in Denmark, Iceland and Norway were 20.4, 27 and 32.8, respectively, per 100,000 persons aged over 50 years [7, 11, 37, 38]. The mean annual incidence in our cohort was 26.7 when we restricted contributing cases to those occurring in the 5-year period (1992–1996) reported in the previous Norwegian studies [11, 37]. A Swedish study covering the time period 1976–1995 reported a similar incidence rate, 22.2 per 100,000 aged over 50 years, but a later study from Sweden (1997–2010) reported a lower average incidence rate of 14.1 per 100 000 aged 50 years or more [29, 36]. A significant increase in biopsy-proven GCA during 1976 through 1995 was described in the former study [36]. In the latter Swedish study, they observed a decrease in the annual incidence rate of GCA from 15.9 in 1997–2001 to 13.3 per 100,000 in 2007–2010 [29]. A 20-year (1990–2009) hospital-based retrospective study from Jerusalem and a 26-years (1986-2012) population-based study from northern Italy also showed significantly decreasing incidence of GCA, the Italian study observing a turning point around 2000–2001 [14, 39]. A hospital-based Spanish study over 25 years showed progressive increase in the incidence until 1996–2000, but a lower rate in the 5-year period thereafter [40]. Chandran et al., reporting results of a large population-based cohort study of Olmsted County in Minnesota, a population with strong Scandinavian heritage, found that the incidence over a 60-year period (1950–2009) steadily increased until 1980, after which the incidence stabilized, albeit not significantly decreased [9]. A British study reported stable incidence of GCA from 1990 to 2001 [12]. These results are similar to that of our study, in which we observed an increase in incidence of GCA throughout the 1970s and 1980s but stabilization of the incidence thereafter. Incidence rates reported in predominantly Caucasian populations of southern Europe, Turkey, Australia and New Zealand are lower than that in our population, ranging from 1.1–12.7 per 100,000 over the age of 50 years [5, 6, 14, 40, 41].

It is worth noting that studies on GCA epidemiology have varied with regards to patient inclusion criteria. In the 1980s there were some studies that also included patients with PMR, and the concept of GCA as a clinical syndrome comprising cranial GCA, large-vessel GCA and PMR has recently had a renaissance [35, 42, 43]. Other studies have narrowed their inclusion to cases verified by biopsy specimens [5, 14, 29]. Chandran et al. and Nesher et al. are the recent studies that allow the most direct comparison to our study [9, 39]. These studies both used ACR 1990 criteria for GCA for the inclusion of patients, though Chandran et al. also included seven patients based on radiological criteria. The publications from Haugeberg and colleagues in 2000-2003 report the main prior epidemiologic studies on GCA in Norway, along with Gran and Myklebust who had published a few years earlier [11, 13, 37].

Recently, Seeliger et al. published results of the Diagnosis and Classification in Vasculitis Study (DCVAS), testing the performance of the ACR 1990 vasculitis criteria [44]. They concluded that since the publication of the criteria, the sensitivity for each type of vasculitis except GCA had diminished, although specificity had remained high. Sensitivity and specificity of the ACR 1990 classification criteria for GCA in the contemporary vasculitis cohort in DCVAS were 81.1% and 94.9%, respectively. Thus, there will be patients who are not identified through the use of ACR criteria for case ascertainment. To minimize potential ascertainment bias caused by this we ensured that patients’ medical records were thoroughly reviewed by a rheumatologist or experienced rheumatology fellow.

Our study supports previous reports about increasing GCA incidence with age, and also that GCA more commonly affects women than men. Greater awareness of GCA among clinicians and altered referral practices from primary care physicians (PCPs) are possible explanations for the observed increase in GCA incidence throughout the 1970s and 1980s. Environmental factors, e.g. the influence of smoking habits and other lifestyle factors, may have contributed to the observed time trends. Ageing of the population with increasing numbers of the old and very old could also be a contributing factor. However, an increase in incidence was seen in all age categories in the earlier time period of our study, reducing the impact of more old people in the population. Furthermore, a Swedish study addressing this possible correlation specifically, concluded that the increase in incidence of GCA in Gothenberg from 1975 to 1995 could not be explained merely in terms of increasing age of the general population [45]. The proportion of patients diagnosed with GCA despite a negative TAB seemed to increase following the publication of new criteria. This could reflect a change over time in both the practice of ordering TAB and the custom to rely on TAB in the diagnostic process.

Our study does have limitations. Retrospective data collection is one of them. The data was extracted from records that were not specifically designed for the study, and there was frequently an absence of data on one or more variables. This may have led us to underestimate the impact of some variables. We particularly emphasize that a large proportion (29.2%) of patients excluded on the grounds of missing records were originally registered with the diagnosis of GCA by the ophthalmology department. This may have caused us to underestimate the frequency of severe ophthalmic manifestations in particular. We also note that the method for measuring ESR changed during the study period. The fact that our cohort is a referral cohort, identified through hospital diagnoses alone, could underestimate the true incidence. Some patients may have been diagnosed by general practitioners or privately practicing specialists and not referred for further investigations, TAB or hospital-based follow up. However, the effect of this case ascertainment bias is most likely small because we rarely identified patients with GCA who were referred in later disease stages. Furthermore, there are very few privately practicing specialists in this region, and those few collaborate closely with the hospitals.

The long duration of our study, 41 years, is a strength giving us the possibility to analyze trends caused by time-dependent factors. However, the long duration also poses a challenge because the diagnostic process might have changed substantially during such a long time period. The use of ACR 1990 classification criteria for inclusion might have caused us to underestimate the incidence by lacking adequate identification of patients with large-vessel GCA. The understanding of GCA has changed substantially during recent years, in particular with regards to large-vessel GCA as a separate phenotype. Our study covers a time period ahead of general awareness of this. For the time period of our study we believe that sufficient and appropriate imaging tests for detection of large-vessel GCA would not have been performed in the majority of cases. This represents a limitation that we share with most previously published studies on GCA incidence. However, our cohort is large and well-characterized following a meticulous chart review, which provided generally complete baseline data.

## Conclusions

Our results are in line with other recent studies on GCA epidemiology demonstrating that the increase in GCA incidence has levelled out. Our results are also consistent with previous studies from Norway demonstrating that our country remains a region with high incidence of GCA in the population. We confirm a persistent increase in incidence with age, and that incidence rates are higher in women than in men. This up-to-date knowledge of the expected GCA disease burden may enable optimized planning of future health care services and prioritization of research domains [16].

## Additional file

Additional file 1: Standardized data collection form (portable document format). (PDF 96 kb)

## Abbreviations

ACR: American College of Rheumatology
CI: Confidence interval
CRP: C-reactive protein
DCVAS: Diagnosis and Classification in Vasculitis Study
ESR: Erythrocyte sedimentation rate
GCA: Giant cell arteritis
ICD: International classification of diseases
PCP: Primary care physician
PMR: Polymyalgia rheumatica
REC: Regional Ethics Committee
RR: Relative risk
SD: Standard deviation
SPSS: Statistical Package for the Social Sciences
TAB: Temporal artery biopsy
