# Correction to: Incidence of giant cell arteritis in Western Norway 1972-2012: a retrospective cohort study

**DOI:** 10.1186/s13075-018-1781-y

**Published:** 2018-12-07

**Authors:** L. K. Brekke, A. P. Diamantopoulos, B.-T. Fevang, J. Aβmus, E. Esperø, C. G. Gjesdal

**Affiliations:** 1 Hospital for Rheumatic Diseases, Haugesund, Norway; 2 Department of Clinical Science, University of Bergen, Bergen, Norway; 3 Bergen Group of Epidemiology and Biomarkers in Rheumatic Disease (BEaBIRD), Department of Rheumatology, Haukeland University Hospital, Bergen, Norway; 4 Martina Hansens Hospital, Bærum, Norway; 5 Centre for Clinical Research, Haukeland University Hospital, Bergen, Norway; 6 Hospital for Rheumatic Diseases (HSRAS), PB 2175, 5504 Haugesund, Norway

**Correction to: Arthritis Res Ther (2017) 19:278**

**https://doi.org/10.1186/s13075-017-1479-6**

Following publication of the original article [1], the authors reported an error. The incorrect sex-specific incidences of GCA were published. The correct mean annual cumulative incidence was 22.0 (95% CI 20.6–23.5) for women and 10.5 (95% CI 9.5–11.5) for men; *p*-value < 0.001 unaffected by the error. Revised versions of Fig. 2 and Table 2 are provided in this correction. Additionally, in the results section (page 4, first section) we report results of a sub-analysis of an extended cohort (*n* = 881). The correct annual cumulative incidence for women in this group was 24.1 and for men 11.6.

**Fig. 2:**
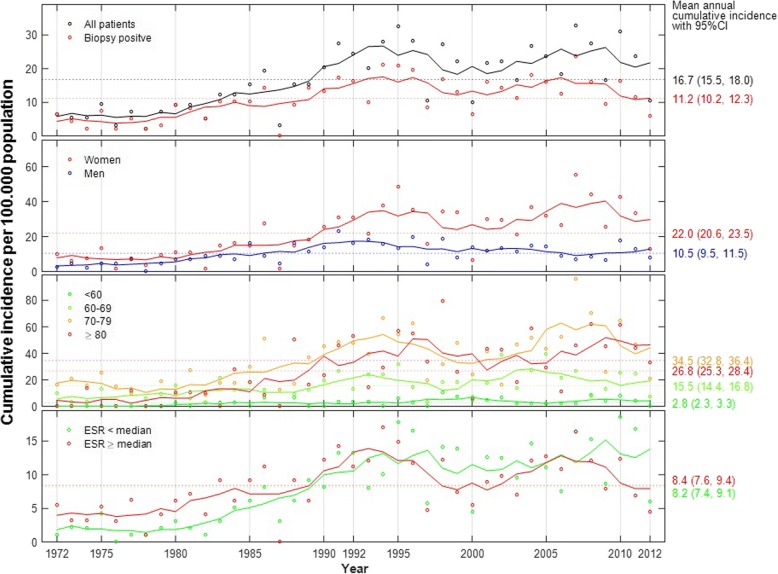
Annual cumulative incidence of giant cell arteritis (American College of Rheumatology (ACR) criteria fulfilled) in Bergen health area 1972–2012. Overall and ESR-specific cumulative incidence calculated as cases per 100,000 general population over the age of 50 years. Incidence by sex was calculated per 100,000 women or men, respectively, and incidence by the different age categories was calculated per 100,000 population of the same age categories (< 60 years, 60–69 years, 70–79 years and 80+ years). Points plotted represent raw incidence. Solid lines were estimated using the smoothing technique of a moving average of 5 years. ESR, erythrocyte sedimentation rate

**Table 2:** The incidence of giant cell arteritis (GCA) in Bergen health area 1972–2012

a Mean annual cumulative incidence						
	All time		1972–1992		1993–2012	
	Cumulative incidence	95% CI	Cumulative incidence	95% CI	Cumulative incidence	95% CI
All patients	16.7	(15.5, 18.0)	11.2	(9.8, 12.7)	22.5	(20.5, 24.7)
Sex						
Female	22.0	(20.6, 23.5)	13.3	(11.8, 14.9)	31.2	(28.8, 33.7)
Male	10.5	(9.5, 11.5)	8.6	(7.4, 9.9)	12.4	(10.9, 14.0)
Age, years						
< 60	2.8	(2.3, 3.3)	1.5	(1.0, 2.1)	4.1	(3.3, 5.0)
60–69	15.5	(14.4, 16.8)	10.9	(9.6, 12.4)	20.3	(18.4, 22.4)
70–79	34.5	(32.8, 36.4)	23.4	(21.4, 25.6)	46.2	(43.3, 49.2)
80+	26.8	(25.3, 28.4)	14.3	(12.8, 16.0)	39.9	(37.2, 42.7)
ESR, mm/hr						
ESR < 85	8.2	(7.3, 9.1)	4.5	(3.7, 5.5)	12.0	(10.6, 13.6)
ESR > 85	8.4	(7.6, 9.3)	6.6	(5.5, 7.7)	10.4	(9.0, 11.8)
b Relative risk (RR) according to time, sex, age and ESR						
	1972–1992			1993–2012		
	RR	95% CI	p-value	RR	95% CI	p-value
Unadjusted						
Time (years)	1.1	(1.1, 1.1)	< 0.001	1.0	(1.0, 1.0)	0.543
Sex						
Time (years)	1.1	(1.1, 1.1)	< 0.001	1.0	(1.0, 1.0)	0.462
Sex (Male vs. Female)	0.6	(0.5, 0.8)	< 0.001	0.4	(0.3, 0.5)	< 0.001
Age						
Time (years)	1.1	(1.1, 1.1)	< 0.001	1.0	(1.0, 1.0)	0.135
60–69 vs. < 60	7.2	(5.1, 10.6)	< 0.001	5.0	(3.9, 6.4)	< 0.001
70–79 vs. < 60	15.4	(11.0, 22.5)	< 0.001	11.3	(9.1, 14.3)	< 0.001
80+ vs. < 60	9.5	(6.7, 13.9)	< 0.001	9.8	(7.8, 12.4)	< 0.001
ESR						
Time (years)	1.1	(1.1, 1.1)	< 0.001	1.0	(1.0, 1.0)	0.632
ESR (>median vs. <median)	1.4	(1.1, 1.9)	0.006	0.9	(0.7, 1.0)	0.116

Overall and ESR-specific cumulative incidence reported as cases per 100,000 background population over the age of 50 years. Incidence for sex reported per 100,000 women or men respectively, and incidence for the different age categories reported per 100,000 population of the same age categories (< 60 years, 60–69 years, 70–79 years and 80+ years). Relative risk calculated according to Poisson regression models for the two timeperiods 1972–1992 and 1993–2012

*ESR* Erythrocyte sedimentation rate

*CI* Confidence interval
