# Supplementary material for: Incidence of giant cell arteritis in Western Norway 1972–2012: a retrospective cohort study
Source: Arthritis Res Ther. 2017 Dec 15;19:278. doi: 10.1186/s13075-017-1479-6 (PMC5732381; doi:10.1186/s13075-017-1479-6)
Supplement: Additional file 1: — Standardized data collection form (portable document format). (PDF 96 kb) [file 13075_2017_1479_MOESM1_ESM.pdf]

Register only information present in the medical record at time of or after first consultation with the diagnosis of GCA.
Insert numerical value or mark the correct alternative answer. If doubt – mark with asterix and supply further details on page 2.

Patient identifier (PID):______ Year of birth: ______Sex (M/F):______ Record review by (initials/date):________________

Time of first registration with ICD-code for GCA: ________ Diagnosed by (unit/type of specialist):__________________________
Year of diagnosis:_________ Year of symptom debut: ___________

ACR-criteria 1990

Criteria		No = 0	Yes = 1	Unsure = 2	Information unavailable = 9
i	Age ≥ 50 years at disease onset				
ii	New onset localized headache				
**iii NB**	Tenderness or …				
**iii NB**	… decreased pulsation in temporal artery				
iv	ESR ≥ 50 mm/hour				
v	Arteritis on temporal artery biopsy				
-	Giant cells in biopsi				

Are the ACR-criteria for GCA fulfilled (≥ 3)	Unsure:	No:	Yes:	End registration if answer is ”No” and ”No”
Is GCA a probable diagnosis in your opinion?	Unsure:	No:	Yes:	

Laboratory results at time of diagnosis (numerical value/unit):

	CRP	ESR	Hemoglobin	WCC	Neutrofils	Eosinofils	Platelets	Creatinin	ALAT
Test result at time of diagnosis (**before** treatment with steroid)									

	Rheumatoid factor	ACPA	ANA	Which ANA-subgroup?	ANCA	Which ANCA sub-group?	Other relevant
Test result at time of diagnosis (**before** treatment with steroid)							

Clinical features at time of diagnosis:

	N = 0	Y = 1	U = 2	I.U. = 9	Comments/further details:
Jaw claudication					
Visual disturbance (related to arteritis)					Subjective:__ Objective:__
Scalp necrosis					
Clinically suspect arteritis outside head/neck					
Hypertension					
Previous cardiovascular disease					Cerebral:__Coronary:__Peripheral:__
Polymyalgia rheumatica					
Peripheral arthritis					
Other relevant:					Diabetes?:__ Smoking?:__

Treatment:
Steroid-treatment at time of diagnosis (name, route of administration and initial dose of chosen steroid):_____________________
Maximum steroid-dose before tapering-regimen (name, route of administration and dose):______________________________

Temporally related	N = 0	Y = 1	U = 2	I.U. = 9	Comments/which specific medication:
Bone-protective treatment following diagnosis?					Calcium/Vitamin D:__ Bisphosphonate:__ PTH-analogue:__ Other:___________
DMARD-treatment following diagnosis?					
Anti-platelet-treatment following diagnosis?					

Outcomes:

	Year – if applicable	N = 0	Y = 1	U = 2	I.U. = 9	Comments/further details:
Vascular/large artery complication?						Thoracic AA:__ Abdominal AA:__ Dissection:_______Other:_________
Death						Cause of death? Sudden death?
Femoral fracture						Low-energy?
Cardiovascular disease – diagnosed after diagnosis of GCA						Cerebral:__Coronary:__Peripheral:__ Aortic valve disease:

Source of recorded information: (mark one or several sources)
Computerized hospital records, Haukeland/Voss ☐
Computerized hospital records, Haraldsplass ☐
Paper files/Central archive ☐

If paper files, from which department(s)? ____
____
____

Other relevant information and comments/further details to information on previous page:
